# A Modified View on Octocorals: *Heteroxenia fuscescens* Nematocysts Are Diverse, Featuring Both an Ancestral and a Novel Type

**DOI:** 10.1371/journal.pone.0031902

**Published:** 2012-02-14

**Authors:** Chen Yoffe, Tamar Lotan, Yehuda Benayhau

**Affiliations:** 1 Department of Zoology, George S. Wise Faculty of Life Sciences, Tel Aviv University, Ramat Aviv, Tel Aviv, Israel; 2 Marine Biology Department, The Leon H. Charney School of Marine Sciences, University of Haifa, Haifa, Israel; Ecole Normale Supérieure de Lyon, France

## Abstract

Cnidarians are characterized by the presence of stinging cells containing nematocysts, a sophisticated injection system targeted mainly at prey-capture and defense. In the anthozoan subclass Octocorallia nematocytes have been considered to exist only in low numbers, to be small, and all of the ancestral atrichous-isorhiza type. This study, in contrast, revealed numerous nematocytes in the octocoral *Heteroxenia fuscescens*. The study demonstrates the applicability of cresyl-violet dye for differential staining and stimulating discharge of the nematocysts. In addition to the atrichous isorhiza-type of nematocysts, a novel type of macrobasic-mastigophore nematocysts was found, featuring a shaft, uniquely comprised of three loops and densely packed arrow-like spines. In contrast to the view that octocorals possess a single type of nematocyst, *Heteroxenia fuscescens* features two distinct types, indicating for the first time the diversification and complexity of nematocysts for Octocorallia.

## Introduction

Within certain epithelial cells of members of the phylum Cnidaria there are distinct intracellular secretion products, known as nematocysts [1], [2]. They are commonly found in the body, and especially in the tentacles, of jellyfish, hydroids, sea anemones, and corals, and are used for capturing food, defense against predation and, in certain cases, for adherence to a substrate [1], [2]. In response to appropriate stimuli, the nematocysts discharge, releasing long, thin, folded tubules, commonly onto a prey where, depending upon the nematocyst type, they either entwine, pierce, or adhere to it [2], [3], [4], [5].

Although nematocysts have been long studied, little is known on those of the anthozoan subclass Octocorallia, most probably due to the generally held view that octocorals possess only a few small nematocysts [6], [7]. To date, most studies report atrichous isorhiza as the sole nematocyst type found in octocorals, as opposed to all other cnidarians, which typically feature several types [8], [9]. Several studies have referred to Mariscal's (1974) review on octocoral nematocysts, and further argued that octocorals generally lack stinging nematocysts, or possess only the atrichous isorhiza type [5], [7]. David et al. [9] indicated that the latter are the ancestral-type for all cnidarians, and the only type present in Octocorallia. A study on *Leptogorgia virgulata* (Alcyonacea) and *Renilla mülleri* (Pennatulacea) indicated the presence of only a very few nematocysts of a single type [6]. Subsequently, Schmidt [10] studied nematocysts in *Alcyonium digitatum*, *Cornularia cornucopiae*, *Parerythropodium coralloides*, *Pseudopterogorgia aerosa* and *Paralcyonium elegans* (Alcyonacea), as well as in *Pteroeides spinosum* and *Veretillum cynomorium* (Pennatulacea), all of which featured solely the atrichous isorhiza type. Schmidt and Moraw [11] examined the nematocysts of *A. digitatum, P. coralloides, P. aerosa* and *V. cynomorium*, concluding that their structure (and possibly also their function) is much simpler than that in the hydrozoans. Sebens and Miles [12] revealed hundreds of nematocysts in the sweeper tentacles of *Erythropodium caribaeorum* (Alcyonacea), characterized as the holotrichous type. Piraino et al. [13] were the only ones to describe two nematocyst types- atrichous and holotrichous isorhiza in *Corallium rubrum*. Jimbo [14] revealed on both the nematocysts and on the zooxanthellae surface in *Sinularia lochmodes* (Alcyonacea), a D-galactose binding lectin (SLL-2), indicating the possibility of a lectin-mediated host-symbiont interaction.

The octocoral *Heteroxenia fuscescens* (Ehrenberg, 1834) is a shallow-water reef-dweller on Eilat's reefs (northern Gulf of Aqaba, Red Sea), where it has been extensively studied, including aspects of its sexual reproduction [15], planula-metamorphosis [16], and acquisition of zooxanthellae [17]. The colonies neither feed on prey nor ingest particulate food, but utilize dissolved organic material and the photosynthates provided by their zooxanthellae [18]. The current study reports for the first time the finding of abundant nematocysts in *H. fuscescens*, including a novel type. These findings shed new light on octocoral nematocysts and challenge the commonly held perception on stinging cells in this group.

## Results

### Cresyl violet stained nematocysts

**Figure 1:**
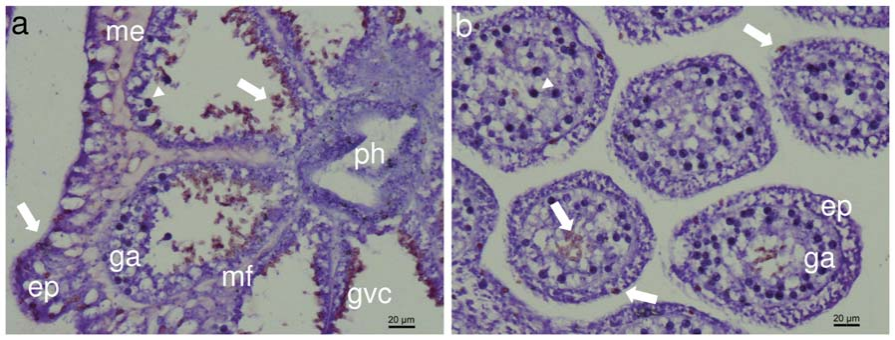
Light microscopy of cresyl-violet stained micrographs of *Heteroxenia fuscescens*. **a**. Cross-section of polyp at pharyngeal level. **b**. Cross-section of tentacular pinnules. Nematocytes stained red (white arrows), pharynx (ph); epidermis (ep); mesoglea (me), gastodermis (ga), mesenterial filament (mf); gastrovascular cavity (gvc), zooxanthellae (white arrow heads).

**Figure 2:**
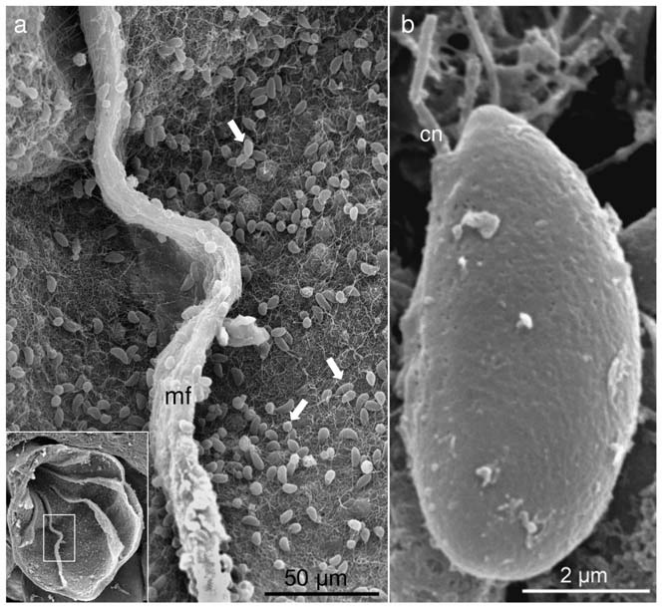
Scanning-electron micrographs of nematocytes within the gastrovascular cavity of *Heteroxenia fuscescens*. **a**. Nematocytes on the gastrodermis (white arrows); insert: cross-section of exposed gastovascular polyp cavity with mesenteries (mf), enlarged area of **a** indicated by rectangle. **b**. Enlarged view of a nematocyte, cnidocil (cn).

**Figure 3:**
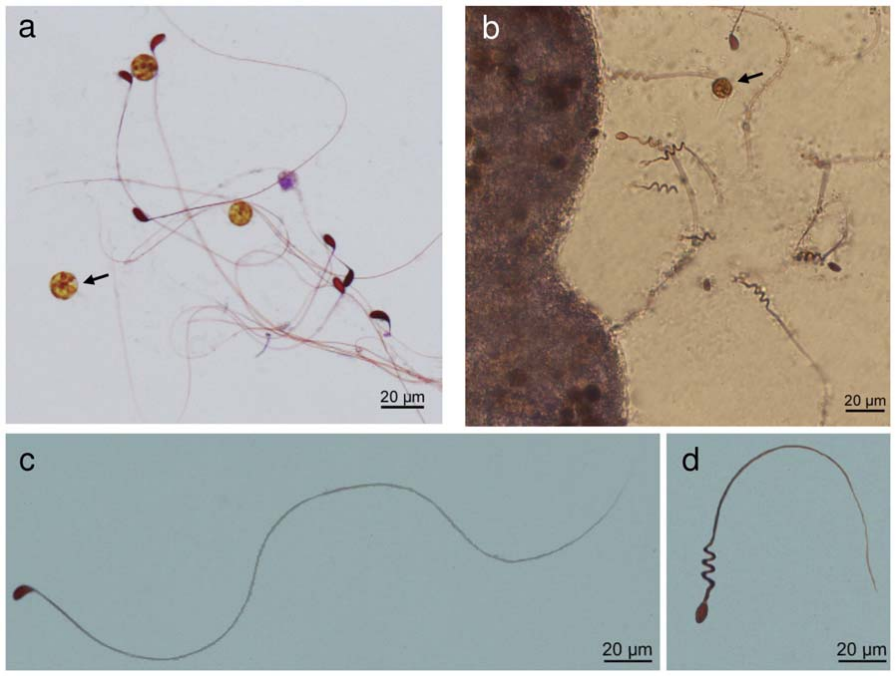
Light microscopy of wet mounts of discharged nematocysts of *Heteroxenia fuscescens* stained by cresyl violet. **a**. Atrichous isorhiza nematocysts isolated from polyp cavity, zooxanthellae indicated by arrow. **b**. Novel type of nematocysts adjacent to surface of tentacular pinnules, zooxanthellae indicated by arrow. **c**. Enlarged view of a discharged atrichous isorhiza nematocyst-type from the gastrovascolar cavity. **d**. Novel macrobasic-mastigophore nematocyst-type from pinnules.

Examination of cresyl-violet stained histological sections of *H. fuscescens* polyps revealed numerous nematocytes (i.e., stinging cells containing the sub-cellular nematocyst), differentially stained red, and thus distinct from the violet-stained epidermis and gastrodermis, pinkish mesoglea, or dark violet zooxanthellae (Fig. 1). The nematocytes were mostly found on the surface of the gastrodermis of the gastrovascular cavity of the polyps (Fig. 1a) and on the tentacular pinnules (Fig. 1b). Notably, the epidermis rarely featured nematocytes (Fig. 1a). In the tentacles the nematocytes mainly appeared within the cavity of the pinnules adjacent to the gastrodermis and less in the epidermis (Fig. 1b). SEM micrographs revealed numerous nematocytes exposed on the gastrodermis, some entangled with the cilia (Fig. 2a). The nematocytes are elongated, about 9 µm in length, have a round and a pointed end, and a laterally-positioned cnidocil (Fig. 2b), which is the mechanosensory cilium receptor for nematocyst discharge [19]. Interestingly, the cresyl-violet- treated wet preparations of the polyp content and tentacular pinnules stimulated massive discharge of nematocyst, which otherwise remained intact. Two types of nematocysts were observed, both red-stained, well differentiated in shape and location. The dominant nematocysts in the gastrovascular cavities feature a capsule, oriented at ∼90° to a uniform long thread tubule (Fig. 3a, c), known to be characteristic to the atrichous isorhiza type [20]. A second, but less common, type was primarily discharged from the tentacular-pinnules. It has a unique thread tubule, with three loops, which narrows toward its distal end (Fig. 3b, d).

### Nematocyst characterization

**Figure 4:**
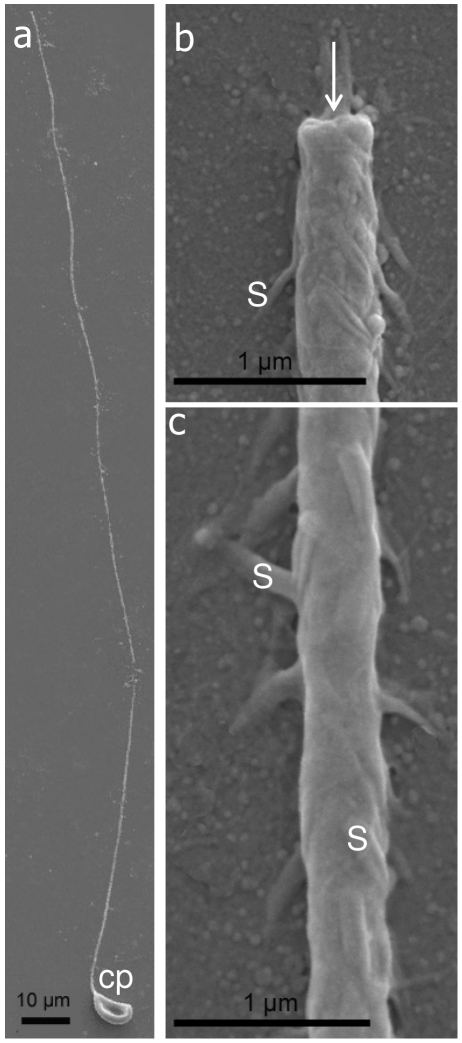
Scanning-electron micrographs of discharged atrichous isorhiza nematocyst of *Heteroxenia fuscescens*. **a**. Whole nematocyst with capsule (cp) and elongated thread tubule. **b**. Enlarged view of the tip (arrow) of the thread tubule with spines (s). **c**. Spines along the thread tubule (s).

The thread tubule of the atrichous isorhiza type, isolated from the gastrovascular cavity, has a uniform diameter along its entire length (Fig. 4a) and an open tip (Fig. 4b). Fine conical spines emerge along the thread tubule, each about 0.5 µm long (Fig. 4b, c). Notably, the spines are indistinct under light microscopy (Fig. 3c), thus corresponding to the definition of atrichous isorhiza nematocysts [8], [21]. The capsule is 9.7±0.75 µm long and 4.3±0.39 µm wide, and the thread tubule is 273.32±55.74 µm long (Table 1).

**Table 1:** Size features of *Heteroxenia fuscescens* nematocysts.

Nematocyst-type	Tube length (µm)	Capsule length (µm)	Capsule width (µm)	Shaft/capsule length
Atrichous Isorhiza (n = 17)	273.32±55.74	9.76±0.75	4.30±0.39	–
macrobasic-mastigophore (n = 15)	136.85±19.68	7.76±0.89	4.61±0.55	4.89±0.61

Size features (mean ±SD) of *Heteroxenia fuscescens* nematocysts: atrichous isorhiza (n = 17) and macrobasic-mastigophore (n = 15). Capsule and tubule length of atrichous isorhiza were significantly longer than those of macrobasic-mastigophore (t-test, *P*<0.01), while the capsule width was similar for the two types. Shaft/capsule length ratio is given for macrobasic-mastigophore nematocysts.

**Figure 5:**
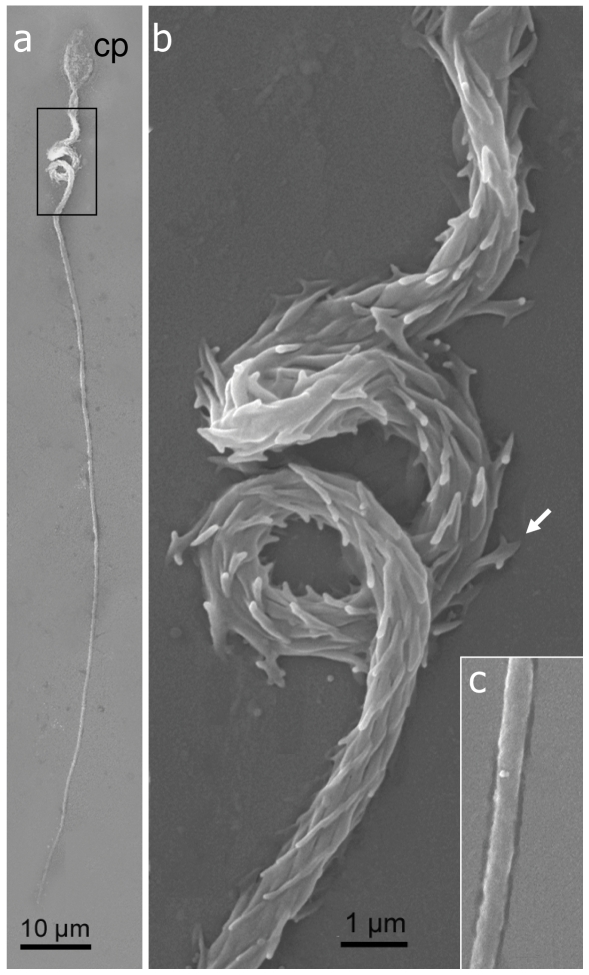
Scanning-electron micrographs of novel macrobasic-mastigophore nematocyst type of *Heteroxenia fuscescens*. **a**. Whole nematocyst with capsule (cp), spiral shaft and elongated thread. **b**. Enlarged view of spiral shaft covered with arrowhead-shaped spines (white arrow). **c**. View of the plain proximal thread tubule. Scale at **b** also applies to **c**.

SEM provided further structural details on the novel type of nematocyst (Fig. 3b, d). It features a basal portion, termed shaft [8], which is spiral and comprises three loops (Fig. 5a). The surface of the shaft features densely packed spines, each ∼1 µm long. These arrow-like spines are each composed of a pair of side-pointed hooks (Fig. 5b). The shaft gradually tapers to a narrower plain thread-tubule without spines (Fig. 5c). The thread-tubule is 136±19.69 µm long (n = 15 nematocysts) and significantly shorter than the atrichous isorhiza-type. Its capsule is 7.77±0.90 µm long, also significantly shorter than the latter, but 4.62±0.56 µm wide, like the atrichous isorhiza (Table 1). To the best of our knowledge this type of nematocyst (Fig. 5) has not been recorded to date in the literature. Since the shaft length is more than four-fold that of the capsule (4.9±0.61, n = 15; Table 1), we have defined the type of nematocyst as macrobasic-mastigophore following [21], [22].

## Discussion

Our preliminary work has indicated that nematocysts of *H. fuscescens* are not stained by the commonly used hematoxylin and eosin dye. Safranin O and methylene blue, known stains of nematocysts, yield non differential results and thus do not distinguish between the different tissue cell-types. Our study indicates for the first time that cresyl violet offers a straightforward staining of nematocysts (“drip and wash”), applicable both to histological sections and live nematocytes. The cresyl violet differentially stained the nematocysts red, in contrast to the rest of the cells (Fig. 1). Moreover, the current study shows for the first time that this dye successfully stimulates discharge of nematocysts both *in vivo* and *ex vivo* (Fig. 3). Cresyl violet is commonly used for neuronal tissue studies, and due to its cationic characteristics it binds readily to anionic macromolecules of the neurons [23]. Thus, we assume that the basic cresyl-violet dye binds to the anionic poly γ glutamic homopolymer inside the nematocyst, releasing cations from the capsule matrix and consequently driving its discharge [24].

The current study in *H. fuscescens* has revealed numerous nematocysts of at least two distinct types: atrichous isorhiza and a novel macrobasic-mastigophore. The former type was mainly found in the gastrovascular cavity, adjacent to the gastrodermis, while the novel one was prevalent in the tentacles. We suggest that the atrichous isorhiza nematocysts play a role in *H. fuscescens* in management of microorganisms introduced with the seawater into the gastrovascular cavity. Whether this is a defense mechanism against parasites and pathogens, as often performed by nematocysts [4], [25], remains to be studied. Interestingly Cheng and Wong [26] described a number of nematocysts of gastrodermal origin that had been discharged into the gastrovascular cavity of the stony coral *Porites* spp., in the vicinity of parasite cysts; and the subsequently found abundance of nematocysts on the surface of the gastrodermis may further support this view. The microstructure of the novel macrobasic-mastigophore-type, and in particular its spiral shaft bearing arrow-like spines (Fig. 4), suggests a specialized protective adaptation whose nature awaits future studies.

The present study has revealed that nematocysts in octocorals are more diversified than previously considered, featuring side-by-side the ancestral atrichous isorhiza and a novel macrobasic-mastigophore. Mastigophore-type nematocysts are found in the most related Hexacorallia sister subclass of Octocorallia, as well as in some other cnidarian groups. It was suggested that the mastigophore-type is in fact an ancestral hexacorallian cnidom and that nematocysts development preceded the diversification of Hexacorallia [27]. The Octocorallia phylogeny places *H. fuscescens* in one of their two major clades, but distant from its suggested root [28], [29]. The Octocorallia subclass is highly diverse, containing over 3,000 species with a remarkably limited nematocyst data-record. It is consequently evident that a thorough study of octocoral nematocytes is still needed. Such a study should also include analysis of the minicollagen proteins, which were shown to vary between simple and more sophisticated nematocysts [9], [30]. This may reveal whether *H. fuscescens* is unique in its subclass, suggesting an independent evolvement of the mastigophores in this species; or, perhaps, that other octocorals also contain this nematocyst-type, thereby not only indicating the existence of a wider array of nematocysts among Octocorallia, but also placing the mastigophore together with the atrichous-isorhiza as an ancestral type of nematocyst [9].

## Materials and Methods

### Specimen collections and maintenance

Colonies of *H. fuscescens* were collected by SCUBA diving in Eilat, shipped to Tel Aviv University, and maintained there in a closed artificial seawater system: seawater temperature ∼24°C, salinity 35–38 ppm, and natural sunlight (2009–2011).

### Light and scanning-electron microscopy

For light and scanning-electron microscopy (SEM) studies, polyps were either removed and preserved in 4% glutaraldehyde in seawater or examined alive. The preserved material was rinsed in double-distilled water (DDW) and decalcified in a mixture of equal volumes of formic acid (50%) and sodium citrate (15%) for 20 min [16]. The polyps then underwent dehydration through a graded series of ethanol and xylene, were embedded in paraffin, and serial cross-sections (5–6 µm thick) were obtained by Shandon MIR microtome. Removal of the paraffin was followed by two short xylene immersions, rehydration with a decreased graded series of ethanol, and a rinse in DDW. Finally, the slides were dried at room temperature, cover slips were glued with Eokkit (03989 Fluka), and the slides were examined under a Nikon OPTIPHOT light microscope.

For SEM studies the decalcified polyps underwent dehydration (see above) and were critically point-dried. The gastrovascular cavity was then exposed with a scalpel blade under a dissecting microscope (Nikon SMZ800), mounted on stubs, and gold-coated. Live nematocysts that were initially stained with cresyl violet (see ahead) were dry-smeared on microscope slides, rinsed with running tap water, dried at room temperature, and then gold-coated. The material was examined under a JEOL JSM 840A SEM (25 kV) and under a Quanta 200 FEG Environmental Scanning Electron Microscope (ESEM) (200 V–30 kV).

### Isolation of live nematocysts

In order to isolate live nematocysts from the gastrovascular cavity of the *H. fuscescens* polyps, a sterile pipette tip (100 µm) was inserted through the mouth opening and the cavity content was removed into Eppendorf tubes. These were centrifuged for five min (600 g),

### Cresyl violet staining

Sections were stained with 0.1% cresyl violet (Sigma C- 1791). The stain was applied by dripping onto the sections, and after two min rinsed under running tap water.

Live nematocysts and wet mounts of tentacular pinnules were rinsed in cresyl-violet before further observation.
